# Purification, crystallization, preliminary X-ray diffraction and molecular-replacement studies of great cormorant (*Phalacrocorax carbo*) haemoglobin

**DOI:** 10.1107/S2053230X14019943

**Published:** 2014-10-25

**Authors:** G. Jagadeesan, P. Malathy, K. Gunasekaran, S. Harikrishna Etti, S. Aravindhan

**Affiliations:** a Department of Physics, Presidency College, Chennai 600 005, India; b CAS in Crystallography and Biophysics, University of Madras, Chennai 600 025, India; c GKM College of Engineering and Technology, Kamaraj Salai, Chennai 600 063, India

**Keywords:** avian haemoglobin, great cormorant, *Phalacrocorax carbo*, molecular replacement

## Abstract

Haemoglobin is the iron-containing oxygen-transport metalloprotein that is present in the red blood cells of all vertebrates. In recent decades, there has been substantial interest in attempting to understand the structural basis and functional diversity of avian haemoglobins. Towards this end, purification, crystallization, preliminary X-ray diffraction and molecular-replacement studies have been carried out on cormorant (*Phalacrocorax carbo*) haemoglobin. Crystals were grown by the hanging-drop vapour-diffusion method using PEG 3350, NaCl and glycerol as precipitants. The crystals belonged to the trigonal system *P*3_1_21, with unit-cell parameters *a* = *b* = 55.64, *c* = 153.38 Å, β = 120.00°; a complete data set was collected to a resolution of 3.5 Å. Matthews coefficient analysis indicated that the crystals contained a half-tetramer in the asymmetric unit.

## Abstract 2

The great cormorant hemoglobin has been isolated, purified and crystallized and the three dimensional structure is solved using molecular replacement technique.

## 1. Introduction   

Haemoglobin (Hb), a well studied globular protein, transports oxygen from the heart to different parts of the body. The physiological function of haemoglobin as an oxygen carrier was first demonstrated by Pfluger in 1875. The three-dimensional structure of haemoglobin is held together by hydrogen bonds, salt bridges and weak noncovalent interactions. Haemoglobin is considered to be an allosteric molecule with oxygen acting as a substrate and protons, chloride ion and organic phosphates acting as allosteric effectors. The oxygen affinity of haemoglobin is expressed by the partial pressure (*P*) of oxygen at which haemoglobin is saturated. In birds, the respiratory system is formed by small air sacs that serve as tidal ventilation for the lungs and have no significant exchange across their cells. The respiratory tract forms a large portion of the total oxygen-storage capacity of the body in birds, whereas in mammals the respiratory-tract oxygen forms a much smaller proportion of the total oxygen storage of the body. Birds are almost unique in their ability to fly, which is a highly energy-consuming form of locomotion. The respiratory system of birds differs from that of mammals by uniquely adapting to very high oxygen consumption during flight. The ability of birds to maintain an efficient oxygen supply to the brain during severe hypoxia is an important adaptation contributing to their exceptional tolerance of extreme altitudes. Compared with mammalian Hb, the presence of hydrophobic residues is increased in avian Hb, which leads to its higher thermal stability and consistent attainment of the tense (T) state (Ajloo *et al.*, 2002 ▶). The conservation of hydrophobic domains in avian Hbs might in fact have been required for the stabilization of tertiary structure in order to maintain the function of the protein through a long period of evolution (Perutz, 1983 ▶).

The great cormorant (*Phalacrocorax carbo*), known as the larger cormorant in India, can be observed fishing even deep underwater and can also fly at high altitude. In general, birds that fly at high altitudes have lower *P*_50_ values; for example, Ruppell’s griffon vulture can fly up to 11 000 m (*P*_50_ = 2.1 kPa), European black vultures fly at about 4500 m (*P*_50_ = 2.8 kPa) and bar-headed geese can fly up to 8000 m (*P*_50_ = 3.6 kPa) above sea level. Cormorant haemoglobin shares nearly 95% sequence similarity with those from Ruppell’s griffon vulture, European black vulture, greylag goose (Liang *et al.*, 2001 ▶) and bar-headed goose (Zhang *et al.*, 1996 ▶). This shows that the cormorant has retained most of the conserved amino-acid residues (Huber *et al.*, 1988 ▶) that help to provide oxygen affinity even at high altitudes. The cormorant can fly at high altitude at a maximum speed of 45.72 km h^−1^ and it can also dive deep into the water to fish even at 30.5–36.6 m. In order to understand the molecular mechanism behind the high oxygen affinity of Hb, we have isolated, purified and crystallized great cormorant haemoglobin and characterized the crystals by means of X-ray diffraction.

## 2. Materials and methods   

### 2.1. Isolation and purification   

**Figure 1:**
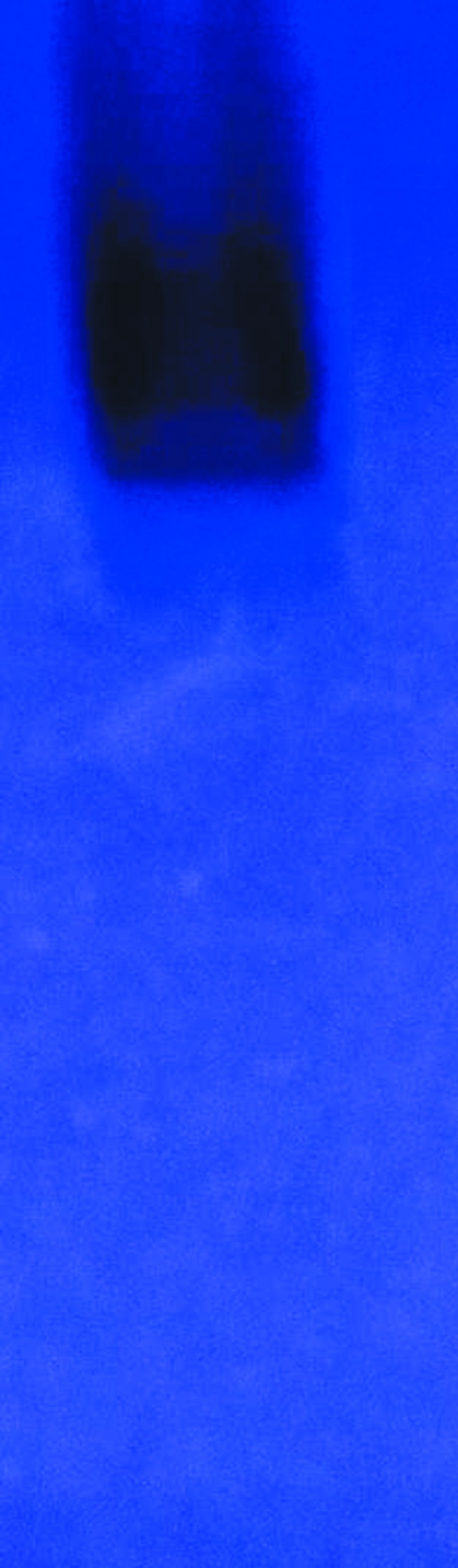
10% native PAGE gel stained with Coomassie Blue. Lane 1, cormorant haemolysate Hb.

Fresh whole blood from great cormorant was collected, transferred immediately to 0.01% EDTA to avoid clotting and stored at 4°C. Red blood cells (RBC) were isolated from blood by centrifugation at 1398*g* for 20 min at 4°C (Neelagandan *et al.*, 2007 ▶). Isolated RBC were washed thrice with two volumes of 0.9%(*w*/*v*) saline solution and haemolyzed by the addition of three volumes of ice-cold Millipore water. Subsequent centrifugation at 5590*g* for 1 h yielded cell-free haemoglobin solution as the supernatant. The isolated protein was extensively dialyzed against distilled water for 24 h to remove trace salts and the sample was then loaded onto a DEAE-cellulose anion-exchange chromatography column (15 × 1.5 cm) equilibrated with 50 m*M* sodium phosphate buffer pH 7. The column was eluted with the same buffer, followed by stepwise elution with various concentrations of sodium chloride (NaCl) solution. A single peak obtained at 0.1 *M* NaCl was collected at a rate of 2 ml min^−1^. A small portion of the sample was used to check for protein content using Bradford assay (Bradford, 1976 ▶) and the purity was assessed by native gel electrophoresis (Laemmli, 1970 ▶; Fig. 1 ▶).

### 2.2. Crystallization and X-ray data collection   

**Figure 2:**
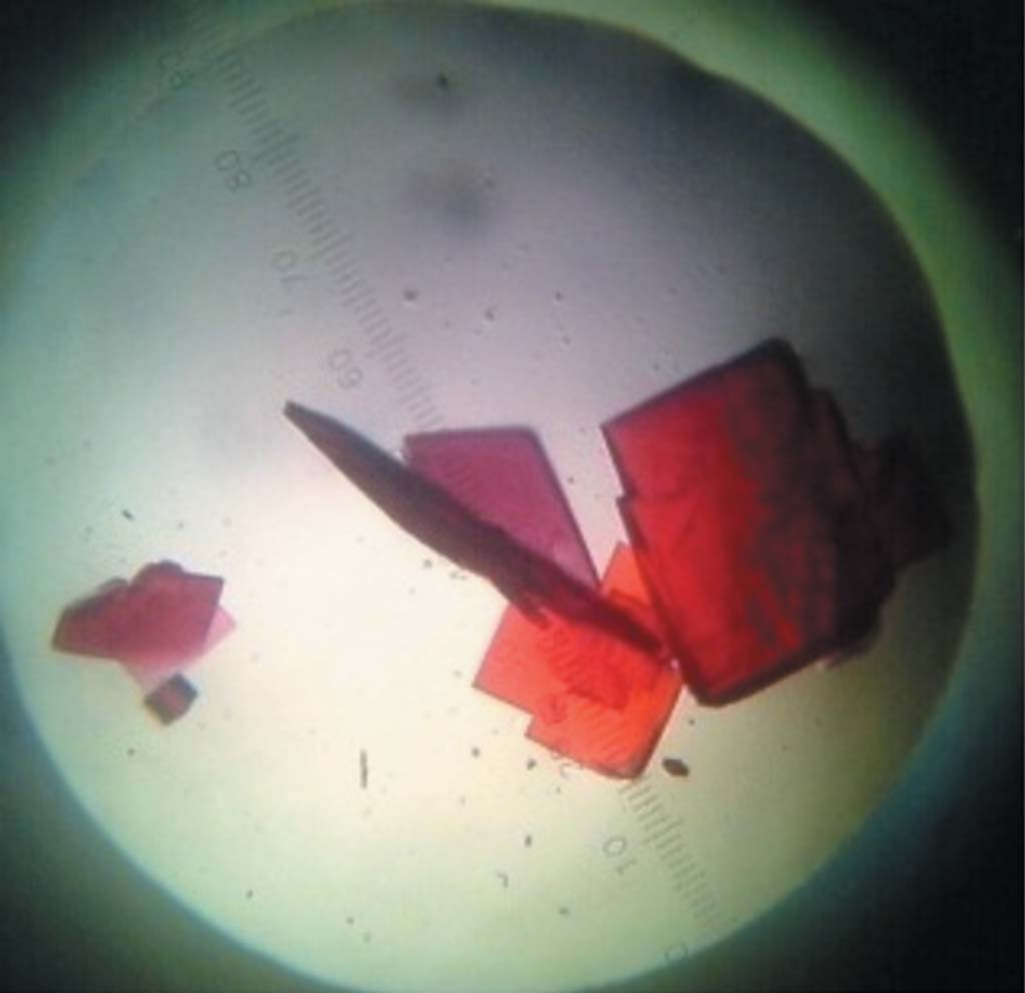
Three-dimensional single crystals of cormorant haemoglobin.

**Figure 3:**
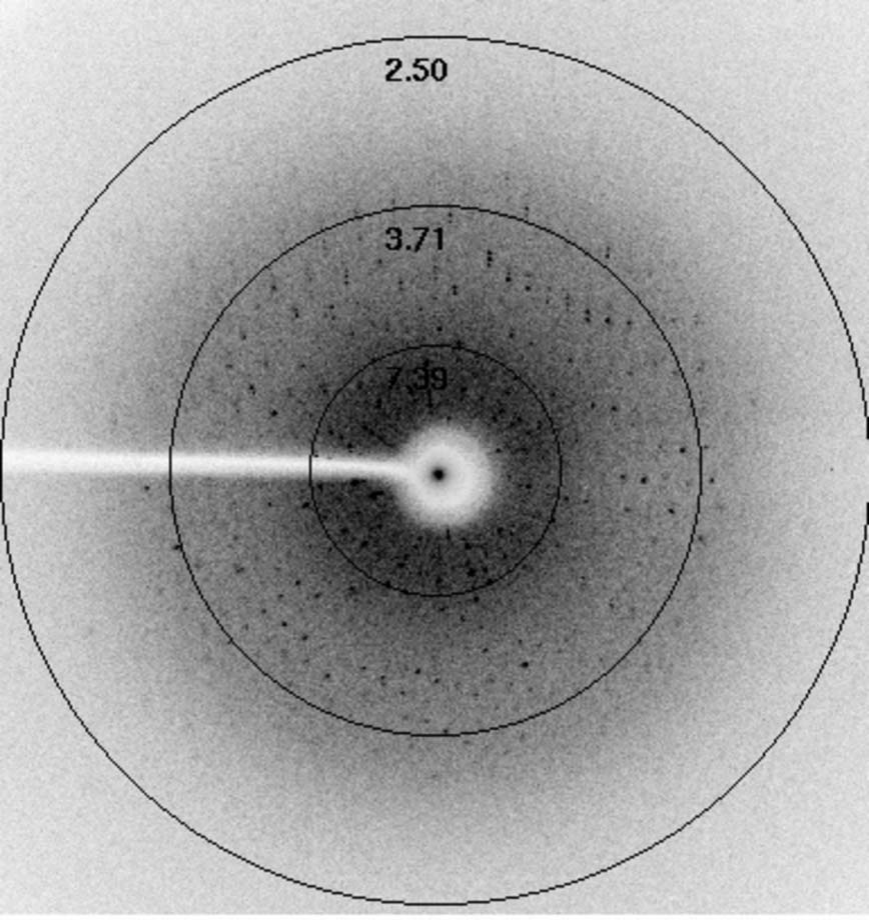
X-ray diffraction pattern of cormorant haemoglobin.

Crystals were obtained by the hanging-drop vapour-diffusion method at 18°C. Polyethylene glycol (PEG) with different molecular weights was initially used to screen the crystallization conditions. It was subsequently found that a combination of PEG 3350 and sodium chloride was suitable for obtaining multiple microcrystal clusters. Single crystals were separated from the microcrystal clusters and immediately flash-cooled in liquid nitrogen, but diffracted poorly with streaky spots at very low resolution. Good crystals suitable for X-ray diffraction were grown after 25 d at 18°C using 25% PEG 3350, 10% glycerol, 0.5 *M* NaCl, 50 m*M* sodium phosphate buffer pH 7.5 equilibrated against 3 µl protein solution and 3 µl reservoir solution (Fig. 2 ▶). The Hb crystals were mounted in a cryoloop and data were collected at cryotemperature using a MAR345 imaging plate at the Central Leather Research Institute (CLRI), Chennai, India. A total of 108 frames were collected at 18°C using a crystal-to-detector distance of 100 mm, an oscillation angle of 1° and an exposure time of 300 s per image; the crystal diffracted to a maximum resolution of 3.5 Å (Fig. 3 ▶). Intensity measurements were processed and analyzed using *iMosflm* (Battye *et al.*, 2011 ▶). The data-collection and refinement statistics are summarized in Table 1 ▶.

## 3. Results and discussion   

Cormorant Hb was crystallized using a slow nucleation process by adding glycerol to the precipitants along with low-salt buffer conditions. Crystals suitable for X-ray diffraction were obtained after 25 d and X-ray data were collected to 3.5 Å resolution. Solvent-content analysis indicated that a half-molecule (α^1^β^1^ subunits) is present in the asymmetric unit with a solvent content of 42% and a Matthews coefficient (Matthews, 1968 ▶) of 2.13 Å^3^ Da^−1^. Attempts were made to solve the structure by the molecular-replacement method using *Phaser* (McCoy *et al.*, 2007 ▶) as implemented in the *CCP*4 suite (Winn *et al.*, 2011 ▶). The amino-acid sequence of both the α and β subunits of cormorant Hb is highly conserved in both bar-headed and greylag goose Hbs. The coordinates of liganded and unliganded goose Hbs were used as initial search models for molecular replacement. Water molecules were removed from the models to avoid model bias and the best solution was obtained using the oxy form of greylag goose Hb (Liang *et al.*, 2001 ▶). Refinement was carried out in *REFMAC* (Murshudov *et al.*, 2011 ▶) as implemented in the *CCP*4 suite. A randomly selected 10% of the total reflections were excluded from refinement in order to use the cross-validation method (Brünger, 1992 ▶). Manual model building and structure validation were carried out in *Coot* (Emsley & Cowtan, 2004 ▶); although the overall resolution of the data set is 3.5 Å only one water molecule was picked up in the β haem site based on a simulated-annealing OMIT map. The final *R*_work_ and *R*_free_ were 0.18 and 0.26, respectively. Further analysis will be carried out to optimize the crystallization conditions to improve the diffraction quality and obtain higher resolution X-ray data in order to understand the molecular mechanism of cormorant Hb.

## Figures and Tables

**Table 1:** X-ray data-collection and refinement statistics

X-ray source	Cu*K*
Wavelength ()	1.5418
Temperature (C)	173
Oscillation ()	1
Exposure time (s)	300
Crystal-to-detector distance (mm)	100
*R*_merge_ † (%)	17.02
Crystal system	Trigonal
Space group	*P*3_1_21
Unit-cell parameters (, )	*a* = *b* = 55.64, *c* = 153.38, = 120
Asymmetric unit content	dimer
Completeness (%)	77.2
No. of unique reflections	2654
*V*_M_ (^3^Da^1^)	2.13
Solvent content (%)	41.68
Resolution limits ()	13.083.5
No. of reflections in test set	282
No. of protein atoms	2217
No. of haem atoms	86
No. of water molecules	01
*R*_work_	0.18
*R*_free_	0.26
Average *B* factor (^2^)	9.4
R.m.s.d., bond lengths ()	0.008
R.m.s.d., bond angles ()	1.250
Ramachandran plot statistics (%)	
Most favoured region	87.2
Additionally allowed region	12.5
Generously allowed region	0.4

† *R*_merge_ = 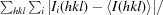 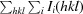, where *I_i_*(*hkl*) is the *i*th measured intensity of reflection *hkl* and *I*(*hkl*) is the mean intensity.
